# Use of Artificial Neural Network Model for Rice Quality Prediction Based on Grain Physical Parameters

**DOI:** 10.3390/foods10123016

**Published:** 2021-12-05

**Authors:** Pedro Sousa Sampaio, Ana Sofia Almeida, Carla Moita Brites

**Affiliations:** 1 Instituto Nacional de Investigação Agrária e Veterinária (INIAV), Av. da República, Quinta do Marquês, 2780-157 Oeiras, Portugal; sofia.almeida@iniav.pt (A.S.A.); carla.brites@iniav.pt (C.M.B.); 2 GREEN-IT Bioresources for Sustainability, ITQB NOVA, Av. da República, 2780-157 Oeiras, Portugal; 3 DREAMS-Centre for Interdisciplinary Development and Research on Environment, Applied Management, and Space, Faculty of Engineering, Lusófona University (ULHT), Campo Grande, 376, 1749-024 Lisbon, Portugal

**Keywords:** artificial neural network, multi-layer perceptron, multiple linear regression, pasting, rice

## Abstract

The main goal of this study was to test the ability of an artificial neural network (ANN) for rice quality prediction based on grain physical parameters and to conduct a comparison with multiple linear regression (MLR) using 66 samples in duplicate. The parameters used for rice quality prediction are related to biochemical composition (starch, amylose, ash, fat, and protein concentration) and pasting parameters (peak viscosity, trough, breakdown, final viscosity, and setback). These parameters were estimated based on grain appearance (length, width, length/width ratio, total whiteness, vitreous whiteness, and chalkiness), and milling yield (husked, milled, head) data. The MLR models were characterized by very low coefficient determination (R^2^ = 0.27–0.96) and a root-mean-square error (RMSE) (0.08–0.56). Meanwhile, the ANN models presented a range for R^2^ = 0.97–0.99, being characterized for R^2^ = 0.98 (training), R^2^ = 0.88 (validation), and R^2^ = 0.90 (testing). According to these results, the ANN algorithms could be used to obtain robust models to predict both biochemical and pasting profiles parameters in a fast and accurate form, which makes them suitable for application to simultaneous qualitative and quantitative analysis of rice quality. Moreover, the ANN prediction method represents a promising approach to estimate several targeted biochemical and viscosity parameters with a fast and clean approach that is interesting to industry and consumers, leading to better assessment of rice classification for authenticity purposes.

## 1. Introduction

With the varying market valorization of rice (*Oryza sativa* L.) production, continuous control of its quality, authentication, and contamination issues is required. Rice quality can be evaluated from grain physical parameters, as well as the milling performance, biochemical composition, and cooking properties [1]. The grain physical parameters include the external and integral properties, such as its appearance (size, shape, smoothness, color), weight, hardness, volume, and flow properties, and are of paramount importance in all activities from harvesting, drying, handling, and storage to milling, packaging, marketing, cooking, product-making, and utilization of rice [1]. In addition to the grain physical parameters, rice quality is characterized by basic chemical composition such as protein, moisture, fat, ash, and amylose content, as well as gelatinization temperature, gel consistency, and pasting viscosity. Amylose content is highlighted due to its correlation with the pasting and retrogradation behavior, influencing the textural properties of cooked rice and the dynamic viscoelasticity of rice starch gel [2]. Proteins and lipids are also parameters currently accepted to define rice quality during processing and storage [3,4]. The acceptability and the commercial value of the paddy according to industrial standards are mostly based on grain milling performance such as the husked, milled, and milled head rice yields. Milling quality aspects are affected by temperature during rice ripening and include chalkiness, immature kernels, kernel dimensions, and fissuring, factors that are related to protein, amylose, and amylopectin chain length [5]. Grain appearance depends on the rice variety and is characterized by grain shape, total whiteness, vitreous whiteness, and chalkiness. These parameters are considered critical factors that affect market acceptability [6]. Rice grain shape is defined by biometric parameters (length, width, length/width ratio), which are used for the designation and classification of the commercial rice types [7,8]. Chalkiness, an opaque white discoloration of the endosperm, reduces the value of head rice kernels and decreases the ratio of head to broken rice obtained during the milling process [9]. Rice varieties with similar grain appearance can reveal different cooking behavior due to their chemical composition, mainly the amylose contents with an impact on the viscosity profiles.

The development of reliable and fast methods for rice quality control has great potential for application in the screening of varieties in breeding programs and the mill industry. The breeding programs and mill industry take advantage of near-infrared and machine learning methods for the classification of the rice varieties and amylose determination [10,11].

Machine learning based on experimental data can optimize grouping and classification, leading to the development of models that can predict the behavior or properties of a specific system [12,13,14], including multiple linear regression (MLR) and artificial neural networks (ANNs).

Multiple linear regression (MLR) and artificial neural network (ANN) models were employed for rice protein prediction using the NIR imaging system and NIR spectroscopy, being considered a non-destructive detection method of rice protein content [15]. Zhang et al. (2012) developed prediction models for rapid monitoring for crude protein content using both ANN and MLR algorithms, presenting significant correlation values (0.92 and 0.90), respectively, for the validation data [16]. The ANN was also considered as an accurate technique to predict the grain yield under different water and nitrogen applications [17]. According to the previous work, this study represents a significant strategy to predict several important pasting and biochemical parameters using the ANN tool based on several biometric data of rice.

MLR is a commonly used algorithm, a prediction tool that determines a mathematical relationship among several random variables to one dependent variable, although no casual mechanism is indicated. An ANN is appropriate in the quality control of several types of food products [18], being considered as a well-known prognostic method used to find a solution when other statistical methods are not applicable. The advantages of the ANN are related to the ability to learn based on examples, fault tolerance, operation in a real-time environment, forecasting nonlinear data, and superior prediction characteristics, making it a widely used statistical tool [19]. Moreover, the ANN accurately fits in the nonlinear variables, which is an advantage compared to multivariate linear analysis [20].

The main objective of this study was to evaluate the ability of a backpropagation ANN and MLR to predict rice biochemical components such as starch, amylose, ash, fat, protein, and pasting parameters (peak viscosity, trough, breakdown, final viscosity, and setback) based on grain appearance (length, width, length/width ratio, total whiteness, vitreous whiteness, and chalkiness) and milling yields (husked, milled, head). This strategy can be considered useful to estimate the biochemical and pasting parameters of rice based on grain appearance and milling yield parameters, providing an important contribution to the rice value chain for industry and consumers and also in the screening of varieties in breeding programs, saving time and decreasing costs associated with the detailed analysis processes.

## 2. Materials and Methods

### 2.1. Rice Sample Preparation

This study, performed in 2021, was based on 66 samples of rice (including *Indica* and *Japonica* varieties) from the Portuguese Rice Breeding Program, whose genotypes (Ariete, OP1001, OP1109, OP1203, OP1212, and Sprint) were grown along the basins of 3 different rivers with very different microclimates (Alcácer do Sal, Salvaterra-de-Magos and Montemor-o-Velho, Portugal) across two seasons (2014–2015). Rice samples were dehusked in a Satake mill (THU, Satake, Taito, Japan) and polished (Suzuki MT98, Santa Cruz do Rio Pardo, São Paulo, Brazil) to assess milling yields and obtain milled (polished) rice.

### 2.2. Milling Yields and Grain Appearance

The potential yields of husked, milled, and head rice were determined according to ISO 6646, 2011 [21]. Biometric parameters of polished rice grains such as length (L), width (W), length/width ratio (L/W), chalkiness (CH), total whiteness (TW), and vitreous whiteness (VW), were evaluated in 50 g samples by image processing (S21 model and software, Suzuki, Brazil).

### 2.3. Biochemical Composition

The polished rice samples were ground using a Cyclone Sample Mill (falling number 3100, Perten, Stockholm, Sweden), with a 0.8 mm screen. Starch (ST), protein (P), fat (FA), and ash (AS) content were assessed using NIR transflection MPA equipment (Bruker Optics, Ettlingen, Germany). The calibrations used were provided by Bruker Company (Billerica, MA, USA). For each sample, approximately 25 cm^3^ of rice flour was loaded in a circular cup and pressed slightly to obtain a similar packing density. Sixteen consecutive scans were performed for a wavenumber range (12,000–4000 cm^−1^), at 16 cm^−1^ resolution. For each rice sample, two spectra were obtained. Amylose (AMY) content was quantified using a standard curve developed from absorbance values of 4 calibrated samples from standard rice varieties (IR8, IR24, IR64, and IR65) obtained from the International Rice Research Institute. The amylose content was determined using a colorimetric technique with a spectrophotometer (Hitachi, Tokyo, Japan) at 720 nm, according to the ISO 6647-2:2015 method [22]. The determination and evaluation of biochemical parameters were performed in duplicate. The value considered is the average of both samples obtained.

### 2.4. Pasting Parameters

The paste gelatinization and viscosity properties of rice were assessed using a viscosity analyzer (RVA-4, Newport Scientific, Warriewood, Australia). Peak viscosity (PV), setback (ST), breakdown (BD), trough (TR), and final viscosity (FV) were determined according to the AACC International Approved Method 61-02.01. The determination and evaluation of physical parameters were performed in duplicate. 

### 2.5. Statistical Analysis and Model Development

#### 2.5.1. Multilinear Regression

Multiple linear regression (MLR) was used to develop a model for predicting the biochemical parameters that characterize the rice grain. MLR is one of the oldest regression methods, being used to establish linear relationships between several independent variables (*Xi*) and the dependent variable (sample property) (*Y*) that depends on them. The model can be represented in the following Equation (1):

(1) $$\;\;\;\;y_{i}=b_{0}+\overset{n}{\underset{i=1}{\sum }}b_{i}x_{i}+e_{i,j}$$

where *y* represents the sample property, *b*_0_ the intercept, *b_i_* represents the computed coefficient for each variable *x_i_*, while *e_i_*_,_*_j_* is the standard estimation error. Each independent variable was analyzed and correlated with the specific property *y_j_*. After the MLR model was developed, the accuracy in the prediction of the dependent variable was evaluated by computation of the correlation coefficient, which is calculated when true values are compared to predicted ones. The determination coefficient (*R*^2^) is one of the most used statistical parameters for the assessment of the developed model regardless of the model type (Equation (2)).

(2) $$R^{2}=\frac{\sum_{i=1}^{n}{\left(\hat{y}-y\right)}^{2}}{\sum_{i=1}^{n}{\left(y-\dddot{y}\right)}^{2}}$$

The statistical analysis of several parameters was performed using the data analysis toolbox in Excel software for ANOVA processing.

#### 2.5.2. Artificial Neural Network (ANN)

**Figure 1:**
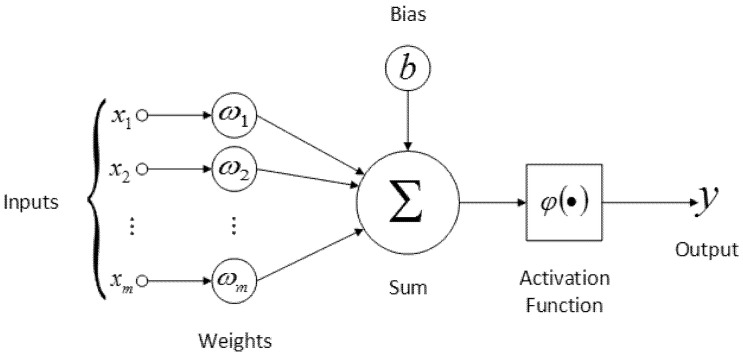
Schematic representation of artificial neural network MLP used for rice biochemical and rheological parameter prediction that consists of three layers of nodes: (1) input layer, (2) hidden layer, and (3) output layer.

The ANN consisted of input and one hidden and one output layer. The number of nodes of the input layer corresponds to the number of variables tested, while the number of neurons in the output layer corresponds to the number of classes. The number of hidden layers and neurons depends on the complexity of the task and the quantity of training data. In the hidden and output layer, each neuron was connected to all the nodes in the proceeding layer by an associated numerical weight. A neural network is an adaptable system that learns relationships from the input and output datasets and predicts a previously unseen dataset of similar characteristics to the input set [23]. A multilayer perceptron (MLP) is a widely used neural network architecture for regression problems, using the backpropagation learning algorithm [24,25,26]. MLPs are usually used for prediction and classification using suitable training algorithms for the network weights (Figure 1). In the ANN models developed, a three-layer network architecture was established, consisting of one input layer, one hidden layer, and one output layer. The input layer accepts the data and the hidden layer processes them, and, finally, the output layer displays the resultant outputs of the model [27,28].

A hyperbolic tangent sigmoid transfer function was used at the input layer and the hidden layer, and a pure line transfer function was used at the output layer. The number of neurons for the input layer is equal to the number of input variables introduced to the networks. According to the biochemical and pasting parameters, the output layer contains one neuron for each parameter in the study. A total of 40 samples out of 66 samples were used for training, and the rest were equally divided for validation and testing (26 samples). Each node, except for the input, is a neuron that is based on a nonlinear activation function. The MLP can be regarded as a hierarchical mathematical function planning some set of input values to output values via many simpler functions. Three different numbers of hidden nodes (4, 8, and 12) were used for the selection of the best models. The multilayer feed-forward fully connected ANN was trained with the Broyden-Fletcher-Goldfarb-Shanno (BFGS) learning algorithm (200 epochs). The number of neurons in the hidden layer was optimized through an early-stop learning procedure. In this procedure, the best topology of the ANNs was searched using the training, validation, and testing datasets. According to the *R*^2^ and *RMSE* values, the best ANN models were developed to predict the different biochemical and rheological parameters. Normally, the nodes are fully linked between layers, and therefore the number of parameters quickly increases with a considerable risk of overfitting [29]. Activation functions of the artificial neurons in hidden layers are necessary for the network to be able to learn nonlinear functions. For the implementation of the backpropagation algorithm, the hyperbolic tangent function was used (tansig). The testing models were verified based on the determination coefficient *R*^2^ and root-mean-square error (*RMSE*). In terms of the model performance analysis, the *RMSE* and *R*^2^ of calibration and validation data were used. The smaller *RMSE* indicates the better performance of models (Equation (3)), where *n* represents the number of the observations in the test data, ŷ is the values of the output in the test data, and *y* represents the value of the predicted output [30]. A significance level of α = 0.05 was used.

(3) $$RMSE=\sqrt{\frac{\sum_{i=1}^{n}{\left(\hat{y}-y\right)}^{2}}{n}}$$

During the ANN development the Levenberg-Marquardt algorithm, derived from Newton’s method, was designed for minimizing functions that are sums of squares of nonlinear functions [31,32]. The Levenberg-Marquardt algorithm is a combination of the gradient descendent rule and the Gauss-Newton method. The algorithm uses a parameter to decide the step size, which takes large values in the first iterations (equivalent to the gradient descent algorithm), and small values in the later stages [33]. The Broyden-Fletcher-Goldfarb-Shanno (BFGS) optimization algorithm, usually used for nonlinear least squares, is presented with the modified backpropagation algorithm, yielding a new fast training multilayer perceptron (MLP) algorithm (BFGS/AG). 

The ANN models were developed using MATLAB^®^ software (R2017a) (MathWorks, Inc; Natick, MA, USA). The MLR models were developed using the Excel application. Ten models were developed separately for predicting biochemical (FA, P, AS, AMY, and ST) and pasting parameters (SB, TR, PV, and FV) based on grain appearance (L, W, L/W, C, TW, and VW) and milling yields (MYH, MYM, and MIY).

## 3. Results and Discussion

### 3.1. Multilinear Regression

**Figure 2:**
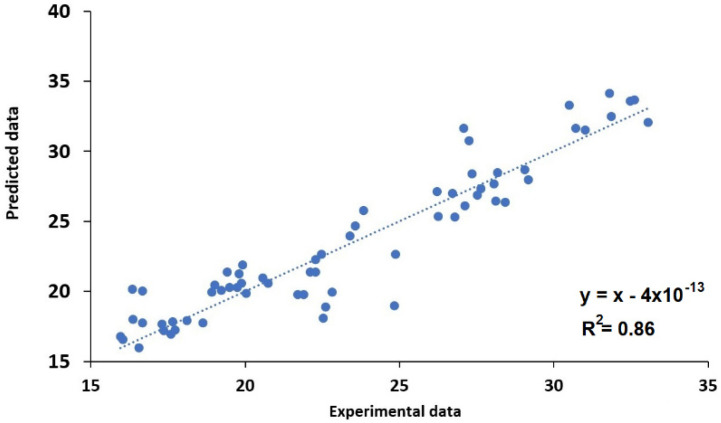
Regression models for biochemical amylose.

The aim of the present study was to evaluate whether the multilinear regression (MLR) technique and artificial neural network (ANN) algorithms could effectively predict rice biochemical and pasting parameters based on the grain appearance and milling yields. MLR models established a relationship between the biological and processing factors and, consequently, the quality feature. The coefficients related to the MLR model, *p*-value, determination coefficient (R^2^), and standard error (SE) of each parameter were determined (Table 1). The *F*-test showed that several independent variables in a multiple linear regression model for all parameters are significant. A low *p*-value (<0.05) represents the high significance of the corresponding coefficient [34]. The SB (R^2^ = 0.92) and AMY (R^2^ = 0.86) are characterized by a significative determination coefficient, which both can be evaluated using a significative predictive MLR model. However, the BD (R^2^ = 0.74), PV (R^2^ = 0.71), TR (R^2^ = 0.62), and ST (R^2^ = 0.62) were characterized by a low determination value and are considered unsuitable for an accurate evaluation of the parameters. The actual experimental data versus predicted values were plotted, showing a relative correlation for BD, PV, and AMY (Figure 2).

However, it was very apparent from the plot that the rest of the models had weaker predictive ability and lower performance (data not shown). Based on these results, the MLR models showed a relative disadvantage because they describe the only linear relationship between variables without considering other types of relations, which can be considered as a limitation.

The detailed analysis of the predictive models for BD, PV, and TR are characterized by a high determination coefficient, in which the MYH and W-white parameters present a positive and significant effect (*p*-value). The acceptability of the paddy according to industrial standards is related to the milling yield, and these parameters can also influence the pasting behavior and their commercial value. Meanwhile, the L-white parameter presents a positive effect in the predictive model of BD and PV but a negative effect in the AMY model. On the other hand, the MYH, L-white, and W-white parameters are characterized by a negative effect in the SB and AMY models. The L/W ratio presents a negative effect compared to the BD and PV and positive for SB, FV, and AMY. The relations between milling parameters and AMY are relevant due to their impact on the cooking behavior of rice, directly affecting the water absorption, firmness of grain, and, conversely, the stickiness of cooked rice [35].

The combined knowledge of the physical properties and anatomical composition of the rice grain is a prerequisite to gaining a deep understanding of what happens to the grain in the different postharvest operations. The understanding of the anatomy of the rice grain explains why rice kernels break so easily on mechanical impact during the physical operations of threshing and milling and under thermal stress during the drying process. The variability between rice grain varieties concerning the surface tissue of the kernel and their layers leads the milling industry to select the correct adjustment of hulling machines to prevent breakage and ensure higher milling recovery. However, it is important to note that there are several correlations among agronomic and quality traits [36,37,38]. Milling quality aspects affected by temperature during rice ripening include chalkiness, immature kernels, kernel dimensions, fissuring, protein content, amylose content, and amylopectin chain length [5].

The TW presents a positive effect in the FV, TR, ST, and FA models but a negative effect in the AS and P models. Studies conducted by Chikubu et al. (1985) found that rice protein content had a negative correlation with appearance, aroma, taste, and stickiness but a positive correlation with hardness [39]. The VW presents a positive effect in terms of the AMY and P predictive models but a negative effect in terms of the FV, TR, and FA models. The FV is an important technological property for the assessment of the cooking quality of rice and paste properties of pre-gelatinized flours. According to Hu et al. (2004), the viscosity profile is a useful parameter in the selection of germplasm with certified quality in rice breeding programs, being subject to varietal differences [40].

The degree of milling or polishing is an important factor that influences the quality of milled rice. Rice milling quality refers to the ability of the kernels to withstand the rigors of hulling and bran removal without breaking, being significantly influenced by genotype, cultural practices, environment, drying, and milling processes [41]. Rice grain quality involves some complex interrelated traits that cover biochemical composition, cooking, eating, nutritional, and sensory properties. Rice endosperm is composed mainly of starch, and its quality is traditionally defined by characterizing starch structure and composition, which is then subsequently correlated with functional properties of the grain [42]. The increase in milling yield may be due to greater agglomeration of starch granules [43], enhancing the endurance of the rice kernels during milling [44]. Milling processes could be influenced by amylose content and starch physicochemical properties. The amylose content in deep milled rice was greater than regular milled rice [45]. Protein composition is also a factor that influences milling performance [46].

The whiteness and gloss of cooked rice are also affected by amylose content [47]. Whiteness, measured with a colorimeter, is used to indicate the degree of milling. However, a common method for the degree of milling quantification is measuring the fat amount on the milled grains. As milling progresses and the degree of milling increases, the whiteness of milled rice increases, the surface lipid content decreases, and milled rice yield decreases. The currently accepted standard for measuring the degree of milling is the Kett Whiteness Meter, and most commercially milled rice must meet some form of degree of milling specification [48].

The whiteness is an important parameter because it is related to the appearance of the grains. The changes of whiteness during milling can be related to their physicochemical properties [49]. Among the factors that influence the percentage of whiteness of the grains, nitrogen fertilization can change the amylose content of the grains [50]. The degree of milling or polishing (e.g., polishing time and polishing pressure) is an important factor that influences the quality of milled rice. The milling operation influences morphological characteristics such as vitreous whiteness, total whiteness, and chalky area vitreous percentage.

Grain appearance is characterized by biometric parameters (length, width, length/width ratio), total whiteness, vitreous whiteness, and chalkiness, being considered as a crucial factor that affects its market acceptability. The L/W ratio is used internationally to describe the shape and class of the rice variety [6]. The CH is characterized by a negative effect on the PV, FV, TR, FA, and ST models, presenting an opposite effect compared to the previous parameters analyzed, which can be related to the specific grain characteristics and, consequently, its effect in biochemical and pasting parameters. Studies performed by Li et al. (2019) showed a correlation between agronomic traits and yield depending on the ecotypes of rice variety [51]. Many studies have shown that the physical characteristics of the rice grain are associated with the yield of head rice [52].

Chalkiness is an undesirable trait that negatively affects milling, cooking, eating, and grain appearance and represents a major problem in many rice-producing areas of the world, being associated with genetic and enzymatic factors. Chalky grains were found to contain a lower density of starch granules as compared to vitreous ones [53].

### 3.2. Artificial Neural Network

The artificial neural network (ANN) was accomplished using the training, validation, and test datasets. Several ANN models were developed using variables related to the grain appearance such as biometrics and milling yield parameters were taken as the input parameters, while protein (P), amylose (AMY), peak viscosity (PV), trough (TR), breakdown (BD), final viscosity (FV), setback (SB), ash (AS), starch (ST), and fat (FA) were considered as output parameters. A multilinear perceptron (MLP) training algorithm was used for ANN model development. The training algorithm and kernel function are very important factors in the ANN. The network structures developed for data included an input layer, one hidden layer, and an output layer. The correlation coefficient between the output and the target simulated value was used to select the optimal number of neurons in the hidden layer. The numbers of neurons in the hidden layers were established when the maximum values of correlation coefficients were found. Three different neuronal structures were tested, characterized by 4, 8, and 12 hidden layers. The input layer (9) and the output layer (1) were similar for all models (9:4:1, 9:8:1, and 9:12:1). According to the results, the best ANN models were characterized by a network model with 12 hidden nodes, presenting high R^2^ for testing step: ST (0.90); AMY (0.94); AS (0.70); P (0.91); BD (0.90); TR (0.93); SB (0.96); PV (0.95); and FV (0.91), while the model for FA is characterized by eight hidden nodes and R^2^ = 0.81. The determination coefficient R^2^ = 0.98 showed a suitable match between the observed and predicted data (Table 2). According to these results, the number of nodes in the hidden layer should be correlated with the quantity of input data. The correlation coefficient, R, between the outputs and targets was a measure of how well the variation in the output was explained by the targets and outputs. The results obtained revealed that the MLP algorithm associated with the Broyden-Fletcher-Goldfarb-Shanno learning algorithm was more efficient in modeling the different biochemical parameters. The neural network can learn complex relationships and generalize results from a specific pattern of data, being considered an appropriate technique for modeling complex systems. Solving problem using ANNs depends on the magnitude, type, quality, and preprocessing step of the training data [54].

#### Model Validation

The model validation was performed based on the R^2^ and RMSE determined with calibration, validation, and test datasets for all parameters (Table 2). The number of hidden neurons was tested to find the best result in term of the correlation coefficient. According to the parameters, the best ANN model was obtained for 12 hidden layers, being characterized by high R^2^ for training, validation, and test models, while the error for each parameter was also very low.

The ANN model improved the estimation results by lowering the value of RMSE compared to the MLR models for the calibration/training set as well as for the validation set, respectively. However, it should be noted that for the calibration dataset, the ANN method always performed better than the MLR method. 

To test the performance of the developed ANN models, the predicted and experimental datasets of training samples were compared, and the results showed the high ability of the ANN to generate outputs close to the experimental data (Table 3). The average accuracy of training data (R^2^ = 0.98) represents that the developed network could be used for testing data in the subsequent analysis. The correlation between the predicted and targets values is highly significant. The average testing accuracy (R^2^ = 0.90) indicates that the developed network is efficient and feasible. The error statistics evaluated for developed ANN models are highly constant for both training and test data of each output, suggesting a lack of overfitting throughput in the training process (Table 3). The important key of the ANN model is not necessary to specify a previous proper fitting function, so it has a complete calculation capability to estimate practically all types of nonlinear functions which helps us to develop the most accurate prediction model. Based on the high accuracy of the predicted data both in the training and testing processes, the neural networks could predict the biochemical and pasting parameters, being fundamental for classification and analysis of rice quality. These achievements are supported by modeling studies previously performed in different research areas that have also indicated higher accuracy of ANN modeling technique than regression modeling [55,56].

However, the promising results, which are following many correlation studies that have been conducted on the relationships among starch quality parameters, can be affected by the wide diversity of rice germplasm and the complexity of the inheritance of quality parameters [57].

**Figure 3:**
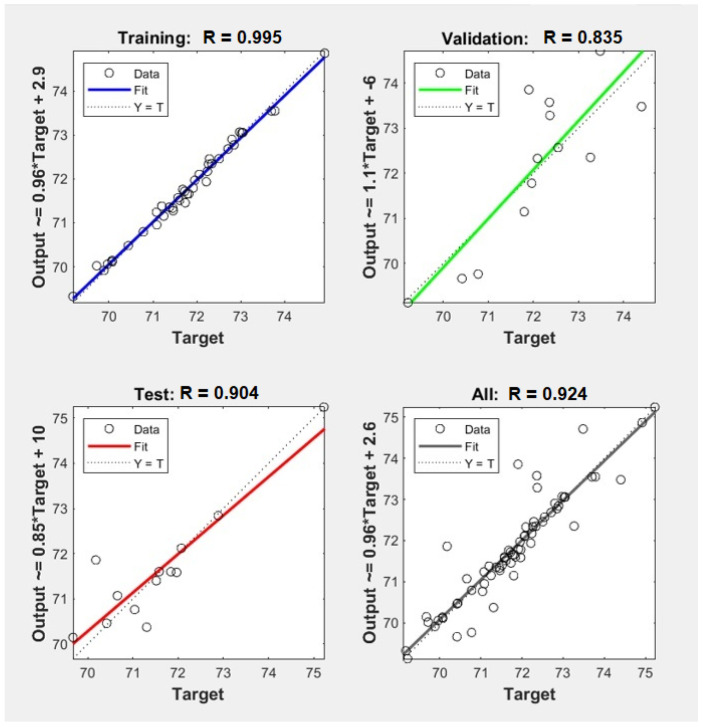
Graphical representation of ANN models associated with each pasting parameter: breakdown (BD).

**Figure 4:**
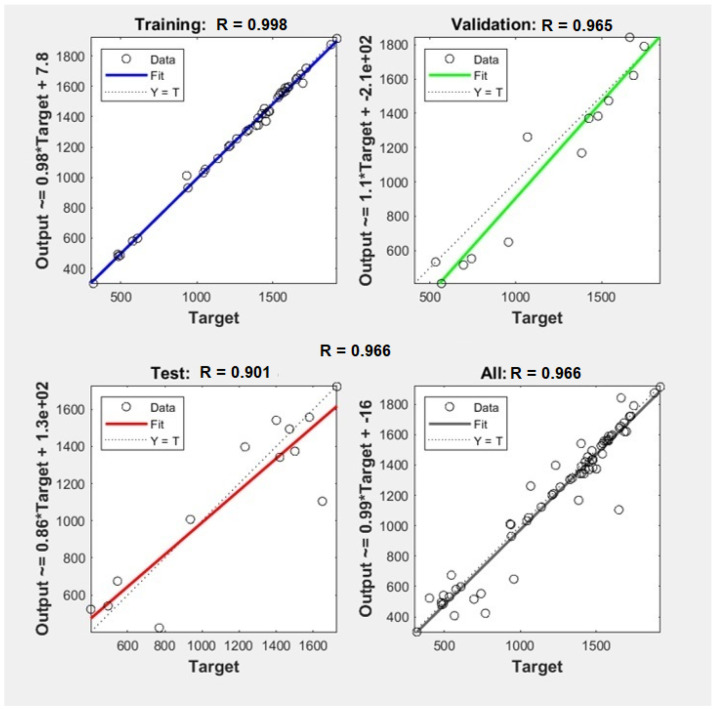
Graphical representation of ANN models associated with biochemical parameters: starch (ST).

As an effective comparison of the prediction models for the parameters, the observed values against the predicted values obtained were plotted, as well as the regression parameters between observed and predicted values, as represented in Table 3 and Figure 3 and Figure 4.

In this study, the MLR and ANN modeling methods were applied for monitoring rice quality using the experimental data registered along with the study. The Figure 3 and Figure 4 show the experimental and predicted values related to the biochemical and pasting ANN models. The ANN models were most efficient, and the regression line between the observed and the predicted values nearly overlapped the 1:1 line, which was the case for both the calibration and the validation sets. High R^2^ and low RMSE values showed that the ANN models present promising potential to improve the estimation of different biochemical and pasting parameters, being especially able to cope with nonlinearity in the dataset. Furthermore, although ANN models are unable to identify sensible bands due to the nature of the method, they resulted in generally higher R^2^ values and lower RMSE values than linear regression models. This implies that the relationship between biochemical and pasting parameters and biometrics properties may indeed be nonlinear. Based on these models, this study showed that the ANN algorithm was an efficient method for biochemical and pasting prediction based on milling yields and grain appearance parameters.

## 4. Conclusions

The ANN algorithms tested in the development of prediction models for rice biochemical and pasting parameters based on grain physical data are characterized by significant regression coefficients. These achievements can be considered as an added value for rice quality improvement in breeding purposes and processing, being suitable for qualitative and quantitative measurement of different physicochemical features of rice. In the future, based on these promissory results, we intend to develop a robust prediction model for several parameters based on a large number of rice varieties from different countries and, consequently, to implement an automatic evaluation system for different pasting and biochemical parameters, reducing costs associated with several time-consuming experimental procedures.

## Figures and Tables

**Table 1:** Comparative analysis of several ANOVA parameters for the models obtained after MLR developed for different biochemical and pasting models developed based on the biometrics and industrial parameters. Peak viscosity (PV); setback (ST); breakdown (BD); trough (TR); peak viscosity (PV); final viscosity (FV); starch (ST); protein (P); fat (FA); ash (AS); amylose (AMY); total whiteness (TW); vitreous whiteness (VW); chalkiness (CH); milling yield husked (MYH); milling yield milled (MYM); milling industrial yield (MIY); length (L); width (W).

	BD		PV		FV		SB		TR		ST		FA		AMY		AS		P	
Parameters	Coefficient	*p*-Value	Coefficient	*p*-Value	Coefficient	*p*-Value	Coefficient	*p*-Value	Coefficient	*p*-Value	Coefficient	*p*-Value	Coefficient	*p*-Value	Coefficient	*p*-Value	Coefficient	*p*-Value	Coefficient	*p*-Value
Intercept	−6111.10	0.050	−8298.4	0.127	−1847.75	0.694	6450.621	0.005	−2187.267	0.410	35.279	0.002	3.543	0.205	89.023	0.003	1.068	0.333	10.2379	0.1625
MYH (%)	133.87	0.000	253.4	0.000	156.14	0.000	−97.304	0.000	119.568	0.000	0.140	0.155	−0.028	0.258	−1.255	0.000	−0.015	0.134	−0.0153	0.8150
MYM (%)	−10.15	0.113	−20.5	0.069	−17.09	0.081	3.413	0.462	−10.359	0.062	−0.045	0.049	0.002	0.753	0.107	0.072	0.001	0.651	−0.0032	0.8307
MIY (%)	−31.06	0.391	−98.6	0.123	−90.54	0.105	8.074	0.760	−67.557	0.034	0.259	0.046	0.007	0.842	−0.096	0.775	0.018	0.168	0.0491	0.5661
L-white (mm)	95.75	0.028	161.3	0.034	76.08	0.245	−85.228	0.008	65.553	0.078	0.069	0.647	−0.025	0.514	−1.327	0.001	−0.025	0.110	−0.1162	0.2517
W-white (mm)	398.83	0.001	648.5	0.003	350.82	0.057	−297.726	0.001	249.711	0.018	0.721	0.090	−0.049	0.649	−3.948	0.001	−0.092	0.035	−0.2333	0.4086
L/W ratio	−533.19	0.000	−701.5	0.001	483.18	0.007	1184.727	0.000	−168.351	0.089	−0.615	0.128	0.094	0.359	9.234	0.000	0.028	0.488	−0.2931	0.2767
TW	34.24	0.412	127.4	0.085	153.66	0.018	26.223	0.390	93.205	0.012	0.357	0.018	0.098	0.011	−0.617	0.112	−0.030	0.046	−0.2783	0.0063
VW	−36.97	0.394	−138.5	0.072	−174.43	0.010	−35.925	0.258	−101.536	0.008	−0.276	0.075	−0.106	0.009	0.815	0.045	0.027	0.083	0.2557	0.0150
CH	−31.84	0.141	−78.0	0.042	−87.72	0.009	−9.682	0.537	−46.197	0.015	−0.285	0.000	−0.057	0.005	0.368	0.067	0.019	0.016	0.1740	0.0011
R^2^	0.74		0.71		0.35		0.92		0.62		0.62		0.33		0.86		0.31		0.27	
R^2^ adjusted	0.70		0.66		0.24		0.90		0.56		0.56		0.22		0.84		0.20		0.16	
Standard Error (SE)	235.62		413.06		359.95		172.21		203.41		0.83		0.21		2.18		0.08		0.56	
*F*-test	1.83 × 10^−13^		3.38 × 10^−12^		0.003		4.4 × 10^−27^		4.30 × 10^−9^		4.11 × 10^−9^		0.01		7.86 × 10^−21^		0.008		0.025	

**Table 2:** Parameters of the ANN models for training, validation, and testing procedures for the biochemical and pasting parameters based on the Broyden-Fletcher-Goldfarb-Shanno (BFGS) optimization algorithm. The transfer function tansig was used along with the model development. Peak viscosity (PV); setback (ST); breakdown (BD); trough (TR); peak viscosity (PV); final viscosity (FV); starch (ST); protein (P); fat (FA); ash (AS); amylose (AMY).

	R^2^			RMSE		
	Training	Validation	Testing	Training	Validation	Testing
ST						
9:4:1	0.91	0.87	0.88	0.250	0.565	0.474
9:8:1	0.91	0.86	0.81	0.119	0.556	2.166
9:12:1	0.99	0.84	0.90	0.015	0.880	0.337
AMY						
9:4:1	0.96	0.94	0.97	2.250	4.35	1.240
9:8:1	0.99	0.95	0.96	0.873	5.78	7.500
9:12:1	0.99	0.94	0.94	0.017	4.09	7.220
AS						
9:4:1	0.90	0.56	0.85	0.002	0.009	0.006
9:8:1	0.92	0.76	0.75	0.001	0.006	0.014
9:12:1	0.94	0.81	0.70	0.001	0.003	0.016
FA						
9:4:1	0.94	0.65	0.62	0.006	0.068	0.184
9:8:1	0.99	0.85	0.81	3.8 × 10^−4^	0.026	0.088
9:12:1	0.99	0.75	0.84	2.03 × 10^−5^	0.088	0.059
P						
9:4:1	0.97	0.84	0.66	0.023	0.190	0.228
9:8:1	0.93	0.75	0.76	0.051	0.177	0.318
9:12:1	0.98	0.82	0.91	0.019	0.470	0.691
BD						
9:4:1	0.97	0.95	0.96	1.1 × 10^4^	2.8 × 10^4^	2.7 × 10^4^
9:8:1	0.99	0.90	0.96	498	4.6 × 10^4^	5.6 × 10^4^
9:12:1	0.99	0.96	0.90	0.001	0.0003	0.0003
TR						
9:4:1	0.96	0.97	0.94	6150	9189	1.7 × 10^4^
9:8:1	0.99	0.95	0.92	1400	9200	2.7 × 10^3^
9:12:1	0.99	0.94	0.93	2736	2.2 × 10^4^	1.1 × 10^4^
SB						
9:4:1	0.97	0.96	0.98	1.6 × 10^4^	3.0 × 10^5^	2.4 × 10^4^
9:8:1	0.99	0.96	0.97	7.1 × 10^3^	3.2 × 10^4^	2.8 × 10^4^
9:12:1	0.99	0.86	0.96	14 × 10^4^	2.2 × 10^4^	5.2 × 10^5^
PV						
9:4:1	0.98	0.92	0.94	1.6 × 10^4^	6.9 × 10^4^	3.4 × 10^4^
9:8:1	0.99	0.98	0.96	3867	3.7 × 10^4^	4.2 × 10^5^
9:12:1	0.99	0.91	0.95	2.6 × 10^4^	1.5 × 10^5^	2.5 × 10^5^
FV						
9:4:1	0.95	0.65	0.91	2.2 × 10^4^	6.7 × 10^4^	3.9 × 10^4^
9:8:1	0.99	0.79	0.78	458	9.5 × 10^4^	4.4 × 10^4^
9:12:1	0.98	0.82	0.91	7.3 × 10^3^	9.2 × 10^4^	3.2 × 10^4^

**Table 3:** Regression statistics parameters describing the relationship between predicted and observed parameters using ANN models.

Parameter	Network	Training			Validation			Test		
		Slope	Intercept	R^2^/RMSE	Slope	Intercept	R^2^/RMSE	Slope	Intercept	R^2^/RMSE
Starch	9:12:1	0.96	2.9	0.99/0.015	1.1	6.0	0.84/0.880	0.85	10	0.90/0.337
Amylose	9:12:1	0.99	0.18	0.99/0.017	0.99	0.36	0.94/4.09	1.2	5.5	0.94/7.220
Ash	9:12:1	0.94	0.035	0.94/0.001	0.84	0.097	0.81/0.003	0.82	0.11	0.70/0.016
Fat	9:8:1	1.00	5.5 × 10^5^	0.99/3.8 × 10^-4^	0.54	0.49	0.85/0.026	0.73	0.28	0.81/0.088
Protein	9:12:1	0.94	170	0.98/0.019	0.76	620	0.82/0.470	0.89	380	0.91/0.691
Breakdown	9:12:1	0.98	7.8	0.99/0.001	1.1	210	0.96/0.0003	0.86	130	0.90/0.0003
Setback	9:12:1	0.96	20	0.99/14 × 10^4^	0.96	−29	0.86/2.2 × 10^4^	1.2	59	0.96/5.2 × 10^5^
Trough	9:12:1	0.89	180	0.99/2736	1.0	−70	0.94/2.3 × 10^4^	0.81	230	0.93/10758
Viscosity	9:12:1	1.00	−51	0.98/7.3 × 10^3^	1.3	910	0.82/9.2 × 10^4^	1.5	1500	0.91/3.2 × 10^4^
Peak Viscosity	9:12:1	0.94	170	0.99/2.6 × 10^4^	0.76	620	0.91/1.5 × 10^5^	0.89	380	0.95/2.5 × 10^5^

## Data Availability

TRACE-RICE—Tracing rice and valorizing side streams along with Mediterranean blockchain.
